# Colloid Silver in Acute Gonorrhea

**Published:** 1907-04

**Authors:** 


### Colloid Silver in Acute Gonorrhea

S. Leon Gans, M.D., Instructor of Genito-Urinary Diseases in the Medico-Chirurgical College, Philadelphia, publishes his extensive experience with colloid silver in acute gonorrhea. (*Medical Bulletin,* February, 1907). From his study of 132 cases he concludes: Instillations of the drug never caused subjective symptoms of irritation. The most striking feature in the entire series was the absence of epididymitis in all cases. There were no complications in any case, unless posterior urethritis is so designated; and this was minimised. All cases ran a much shorter course than under other plans of treatment. The long stage of decline was conspicuous by its absence. There was no sudden cessation of the acute discharge followed by a persistent mucoid exudate, as with many cases treated by the old irrigation methods.

Three to five per cent. collargolum solutions with mucilage sassafras med., being preferable to watery solutions, were injected as soon as the patients presented themselves. The instillation is made with the greatest care and gentleness four times a day, immediately after urination, the instillator being placed just within the compressor urethræ muscle. This insures medication of the infected membranous and prostatic urethra; if introduced farther the instillator deposits the fluid on the base of the bladder, which is rarely at fault. The solution is held five minutes, the meatus being compressed laterally. The urine should be watched, and persistent turbidity not due to phosphates or urates is an indication to decrease the strength of the solution. Number and concentration of the injections should be adapted to the individual needs.

Thirty minutes after a five per cent. collargolum solution had been thrown in and held five minutes, a normal urethra was examined with a straight tube endoscope with an electric bulb. The mucous membrane was found to be stained brown and to have a peculiar lustre, and small areas of normal tissues were apparent at irregular intervals. The author appends a table which shows the characteristic effects of collargolum in twelve typical cases taken at random.

